# Disposable Duodenoscopes: Evidence and Open Issues

**DOI:** 10.3390/life13081694

**Published:** 2023-08-05

**Authors:** Clara Benedetta Conti, Fabrizio Cereatti, Raffaele Salerno, Roberto Grassia, Miki Scaravaglio, Carmen Laurenza, Marco Emilio Dinelli

**Affiliations:** 1 Interventional Endoscopy Unit, Istituto di Ricovero e Cura a Carattere Scientifico (IRCCS) San Gerardo dei Tintori, Via GB Pergolesi 33, 20900 Monza, Italy; 2 Gastroenterology and Digestive Endoscopy Unit, Ospedale dei Castelli, 00040 Ariccia, Italy; 3 Division of Gastroenterology, Azienda Socio-Sanitaria Territoriale (ASST) Fatebenefratelli Sacco, 20121 Milano, Italy; 4 Gastroenterology and Digestive Endoscopy Unit, Azienda Socio-Sanitaria Territoriale (ASST) Cremona, 26100 Cremona, Italy

**Keywords:** single-use duodenoscopes, endoscopic retrograde cholangiopancreatography, economic impact, environmental impact, cap disposable duodenoscopes

## Abstract

Duodenoscope-related infections are a major concern in medicine and GI endoscopy, especially in fragile patients. Disposable duodenoscopes seem to be the right tool to minimize the problem: a good choice for patients with many comorbidities or with a high risk of carrying multidrug resistant bacteria. Urgent endoscopy could also be a good setting for the use of single-use duodenoscopes, especially when the risk of the infection cannot be evaluated. Their safety and efficacy in performing ERCP has been proven in many studies. However, randomized clinical trials and comparative large studies with reusable scopes are lacking. Moreover, the present early stage of their introduction on the market does not allow a large economical evaluation for each health system. Thus, accurate economical and safety comparisons with cap-disposable duodenoscopes are needed. Moreover, the environmental impact of single-use duodenoscopes should be carefully evaluated, considering the ongoing climate change. In conclusion, definitive guidelines are needed to choose wisely the appropriate patients for ERCP with disposable duodenoscopes as the complete switch to single-use duodenoscopes seems to be difficult, to date. Many issues are still open, and they need to be carefully evaluated in further, larger studies.

## 1. Introduction

**Figure 1:**
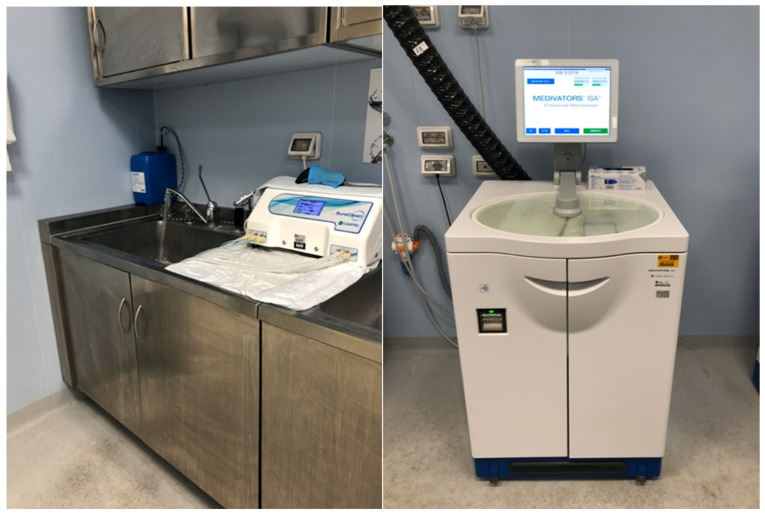
High-level disinfection and reprocessing machine for endoscopy.

During the past decade, there has been an increased recognition of duodenoscope-associated infections related to multidrug-resistant organisms (MDROs), especially Pseudomonas aeruginosa and carbapenem-resistant Enterobacteriaceae (CRE) [1,2]. A 2018 nationwide European study sampled duodenoscopes. Contamination was defined as any microorganism with ≥20 colony-forming units (CFU)/20 mL and the presence of microorganisms with gastrointestinal or oral origin independent of the CFU count (MGO). Overall, 37% of the duodenoscopes were contaminated, and 15% of them were contaminated with MGOs. The study concluded that the reprocessing and process control procedures were not adequate [3]. In 2019, preliminary results revealed duodenoscope contamination rates of 3.6% and 5.4% for low-to-moderate and high concern organisms, respectively, despite the adherence to the recommended reprocessing guidelines. In 2020, a large meta-analysis showed that 15% of duodenoscopes harbored MGOs independent of the CFU count [4]. High-level disinfection (HLD) (Figure 1) eliminates pathogens only partially, and even the double cycle of HLD seems to be ineffective [5,6]. However, HLD is not the only factor that affects the likelihood of infection: the complex design of duodenoscopes, including the elevator, water and the air channels, does not allow them to be completely cleaned, and, today, this is recognized as one of the main problems. Additionally, the creation of a biofilm into the scope channel can reduce HLD effectiveness [7]. The patients and their clinical conditions are also part of the factors that contribute to the transmission of MDROs, especially in the presence of immunosuppression, multi-antibiotic therapies and many comorbidities [3,8,9,10]. Moreover, the recent COVID-19 pandemic has driven the world’s attention to the importance of reducing virus transmission from one patient to another patient and from patients to their healthcare workers.

The US Food and Drug Administration (FDA) has encouraged research into new duodenoscope designs to improve or eliminate reprocessing and to enhance safety [5].

Therefore, efforts in reducing the problem of duodenoscope-related infections have led to the production of single-use, fully disposable duodenoscopes (SUD). Today, two single-use duodenoscopes are available on the market: Boston Scientific’s EXALT Model D and the Ambu A/S Ambu aScope Duodeno (Table 1). The safety and performance of disposable duodenoscopes have been evaluated in many studies. A meta-analysis compared three models of reusable duodenoscopes with disposable ones, focusing on the possibility to perform complete endoscopic retrograde cholangiopancreatography (ERCP), stent placement/removal and stone extraction with a balloon or basket. Image quality ratings were lower for the one reusable duodenoscope compared with the SUD and the two other reusable duodenoscopes (median: 8.0 vs. 9.0, 9.0 and 9.0; *p* < 0.01) [11]. However, many other studies proved that disposable duodenoscopes have greater efficiency and safety comparable to reusable ones. Muthusamy et al. and Slivka et al. measured the feasibility, safety and performance of SUDs using the EXALT Model D SUD across all four American Society for Gastrointestinal Endoscopy (ASGE) ERCP complexity grades, switching from an SUD to a reusable duodenoscope [12]. The ERCPs of the study belong to all ASGE grades: grade 1 (least complex; 7 patients [11.7%]), grade 2 (26 patients [43.3%]), grade 3 (26 patients [43.3%]) and grade 4 (most complex; 1 patient [1.7%]). Fifty-eight ERCPs (96.7%) were completed using the SUD only, and two ERCPs (3.3%) were completed using the SUD, followed by crossover to a reusable duodenoscope. Of the two cases requiring crossover, only one was successfully performed with a reusable duodenoscope. [12]. Also, Napoléon et al. reported a similar procedure success rate of 95% with SUD [13]. Overall, in the available studies, the SUD was rated as comparable to a reusable duodenoscope for 97.9% of all performance metrics measured.

Therefore, the need of reducing duodenoscope-related infection is mandatory and both the safety and the performance of the disposable duodenoscopes seem to be good, even if larger studies are needed. Starting from these data, the present review focuses on the available evidence and the open issues of the future large use of SUD. We describe the possible indications, the economic and environmental implications of future large use of disposable duodenoscopes and we briefly summarize the possible alternative tools.

## 2. Materials and Methods

The present manuscript is a review of the literature. We performed systematic research in PubMed, Medline and Embase databases using the terms “disposable duodenoscope”; “single use duodenoscope”; “reprocessing”; “high level disinfection”; “disposable cap duodenoscope”; “environmental impact AND endoscopy”; “economic impact AND endoscopy”; “Multidrug-Resistant Organisms AND endoscopy”; “COVID-19 AND duodenoscopes”; “COVID-19 AND endoscopy”; “infections AND endoscopy”; “duodenoscope”, and “ERCP AND infections” and we selected review and original articles written in English.

## 3. Results

### 3.1. Indications for the Use of Disposable Duodenoscopes

The appropriate use of SUD is a new topic and evidence is very limited. Indeed, guidelines are missing. Even if the data are few, we tried to identify different categories of factors that could be considered for a selected use of the disposable duodenoscopes in most hospitals.

#### 3.1.1. Patient-Related Factors

Considering that the duodenoscopes-related infections can be linked to the transfer of the bacteria from patient to patient through the duodenoscope, the SUD could be addressed to the patients that need to be protected from MDRO. Furthermore, the use of disposable duodenoscopes has been endorsed during the COVID-19 pandemic to reduce patient-to-patient and patient-to-personnel transmission of the virus, bypassing the reprocessing limitations. However, relevant data on the use of SUD during the COVID-19 pandemic are missing, probably because their costs limited the wide spread of these technologies during the pandemic.

There are some categories of fragile patients prone to develop biliary infections or infections in general, such as immunocompromised patients: transplanted ones or patients on immunosuppressive drugs. In detail, biliary complications are the leading causes of morbidity and mortality after liver transplant (LT). Yang et al. investigated a total of 372,814 hospitalizations occurred in LT patients between 2007 and 2017. ERCP was performed in 2.05% (n = 7632) of all hospitalizations. There was a rise in ERCP procedures from 1.96% (n = 477) in 2007 to 2.05% (n = 845) in 2017. Among LT patients who underwent ERCP, the in-hospital mortality rate was 1% (n = 73). Overall, 8% (n = 607) were discharged to facilities. The mean length of hospital stays was 7 ± 0.3 days. Septicemia was the most common periprocedural complication (18.3%, n = 1399) followed by post-ERCP pancreatitis (8.8%, n = 674) [14]. In a prospective cohort study conducted on 744 patients that underwent 1401 ERCP, the antibiotic susceptibility profiles of bacteria in bile samples and its clinical relevance were investigated [15]. The bile samples of immunocompromised patients (end-stage kidney disease, end-stage liver disease or malignancy, pharmacological immunosuppression) significantly differ from those belonging to immunocompetent patients. This was more evident for fungobilia, which was detected almost exclusively in samples coming from immunosuppressed patients. Particularly, the incidence of Candida in bile was significantly higher in these group of patients. ERCP procedures have been increasing among LT patients. ERCP indications in these patients include dilation and stent placement of anastomotic or non-anastomotic strictures, diagnosing of post-operative biliary leakage, management of papillary stenosis and removal of bile duct stones. ASGE recommends routine antibiotic prophylaxis prior to all ERCPs in LT patients [16]. Importantly, the pathogen detected in these patients after the endoscopy, especially if they receive multiple ERCP, could also come from the translocation from the GI tract of the patient itself to the biliary tree. Thus, in these population, the available data support the practice of peri-procedural antibiotic also to prevent the bacterial translocation from the patient’s own GI tract.

However, these recommendations are based on conflicting data, with post hoc sub-group analyses and inconsistent follow up [17,18]. Moreover, the antibiotic prophylaxis may increase the risk of multi-drug resistant organisms and antibiotic resistance itself, especially to fluoroquinolone. Kohli et al. evaluated LT patients who underwent elective, outpatient ERCP from 2008 and 2015 in a retrospective case–cohort study They assessed the utility of antibiotics in a subset of LT hypothesized as low risk group: LT patients without cholangitis, free from biliary strictures or recurrent primary sclerosing cholangitis. In this group, the authors documented a very low risk of clinically significant ERCP-related infection (0.5%). Furthermore, 109 ERCPs (46%) were performed without antibiotic prophylaxis and none of these patients reported a clinically significant infection after the procedure [19,20]. Nevertheless, the study did not consider long hospitalized patients and those with incomplete biliary drainage, recurrent PSC after LT, and ischemic strictures.

Therefore, immunosuppressed patients and above all LT patients, especially when belonging to high-risk group, could be a target population for the SUD use.

Few studies have compared the rates of ERCP adverse events in those with and without PSC [21]. A systematic review and meta-analysis [22] found a significant increase in cholangitis after ERCP in PSC group, compared to patients without PSC (OR 3.263, 95% CI 1.076–9.896; *p* = 0.037). This increase in infection was observed despite the consistent antibiotic use in most studies, in agreement with previous research, that reported difficulties in the clearance of bacterial overgrowth in PSC patients [23]. In addition, the antibiotic type, the route of administration, and the length of the therapy varies considerably among the studies. Currently, there are no randomized studies that indicate the optimal antibiotic treatment [24]. These data highlight the need to determine the best strategies to reduce the risk of cholangitis in this high-risk population, such as the use of disposable duodenoscope.

On the other hand, the reusable duodenoscopes themselves should be kept free from the risk of being infected by patients at risk of carrying MDROs or viruses, such as COVID-19. Some factors could be identified as predictors of high risk of carrying MDROs. The independent factors that increase the patient’s risk of becoming infected with CRE are prior treatment with carbapenems or another antibiotic (e.g., fluoroquinolones and broad-spectrum cephalosporins); receiving treatment in an intensive care unit; having received mechanical ventilation; being elderly; immunosuppression status; carrying a central venous catheter; and diabetes [25]. Hospitalized patients receiving medical treatment, long-term acute care facilities, and nursing homes are reported as more prone to develop CRE infections. However, according to the Centers for Disease Control and Prevention (CDC), CRE will soon be responsible for infections in community settings too [26]. In 2013, the USA reported the largest outbreak of CRE in his history. Overall, 38 hospitalized patients were found to be either colonized (n = 28) or infected (n = 10) with CRE following ERCP. However, six other patients (forty-four patients, in total) were also found similarly infected or colonized with CRE at the same time, although none of them had received care at the hospital where the other cases occurred. It was unclear if some of the six patients had been in contact with the 38 affected patients and CDC opened a large investigation without reaching a definitive explanation. Therefore, community-associated transmission of CRE could also be another point to take into consideration [25].

#### 3.1.2. Urgent Setting

ERCP is the gold standard procedure for biliary emergencies such as acute cholangitis and urgent biliary drainage. These procedures are frequently organized also at night or during the weekend, when the cleaning and reprocessing staff and materials are in a shorter supply, compared to an elective procedure. Prat et al. focused on the SUD use for emergent procedures in a real-life setting, without the possibility of switching to a regular scope. SUDs were found to be associated with a high rate of procedure completion, similar to what it would have been expected with reusable scopes. Overall, 19 out of 21 procedures (90%) were successfully performed. ASGE procedures grades were 1, 2, 3, and 4 in 14%, 43%, 29%, and 14% of procedures, respectively. Some reported issues were the pushability and the imprecise image, that was often reported as skewed toward yellow tones in few procedures [27]. Nevertheless, the possibility of performing good ERCP in an urgent setting with disposable duodenoscopes is very important. Indeed, it would be much more practical and, importantly, many patients requiring ERCP in urgent settings are often fragile and/or possible CRE carriers.

#### 3.1.3. Hospital Factors

The only available guideline for sampling and culturing of duodenoscopes is a multi-society guideline of the CDC, the FDA and the ASM [28] that contains over 100 steps, making it very difficult and time consuming for a wide application. Moreover, few hospitals have a systematic program of sampling and even when applicated, it cannot be enough to prevent contamination. The single-use duodenoscope could be a good alternative in those hospitals with a higher MDRO infection rate than the national average and where the systematic sampling of scopes is very difficult to realize.

### 3.2. Cost Effectiveness of Single-Use Duodenoscopes

The overall direct and indirect costs of a procedure are difficult to be evaluated. Indeed, they may vary according to the economic system of each country. In USA, the retail prices of SUD ranges from USD 1995 to 4400 [29], but its price may greatly vary according to the different areas, hospital volumes and individual negotiations or discounts. A recent study calculated that the per-procedure cost of SUD in the USA can vary from USD 797 to 1547 for high volume centers and from USD 1318 to 2068 for low volume centers [30]. Moreover, the literature that specifically evaluates the costs of duodenoscopes-related nosocomial infection is limited. This makes it hard to properly understand the economic benefits of using SUDs. Nonetheless, the available data assessed that the average “added” cost for each nosocomial infection ranges from USD 11,000 to 16,500 [30,31]. The studies tried to evaluate the economical implication of the transition to SUDs, calculating the per-procedure overall cost of ERCP with SUDs. The authors created a financial model accounting for the overall costs of duodenoscopes, maintenance, repair, reprocessing supplies, scope washer, man-labor as well as medical “added” expenses of a nosocomial infection. The rate of duodenoscopes-related nosocomial infection was estimated at around 0.4–1%. The costs related to litigation, malpractice claims, bad reputation and loss of patients was not evaluated due to the difficulties in properly quantifying them. According to this model, a “break even” cost for the transition to SUDs was evaluated even according to the ERCPs volume. For high-volume centers (>150 ERCPs/year), the “break even” cost for SUD was >USD 800, whereas for low volume centres (<50 ERCPs/year) was >USD 1300. The authors concluded that, for the large volume centers, SUDs should be priced much lower to break even on costs [31].

Das et al. [32] performed a cost-effective analysis of SUDs with Exalt Model D using a Markov model to compare SUDs with other approaches used to reduce the risk of duodenoscopes contamination. The costs of SUDs were compared with the following reprocessing techniques: standard recommended reprocessing, culture and quarantine approach (culture and 48 h of quarantine before re-use), ethylene oxide reprocessing. The authors concluded that Exalt Model D is a viable and cost-effective strategy for standard ERCP and that its use should be implemented in daily practice. Nonetheless, the study has some major limitations that hampers its conclusions. Firstly, their approach is viable only in the United States in which specific reimbursements are applied for the new technologies. Secondly, the study did not take into consideration the newly designed duodenoscopes with disposable distal ends.

A recently published paper [33] assessed the cost-utility of different approaches to minimize the infection transmission. Interestingly, the authors included even the newly designed disposable distal end duodenoscopes. Using a Monte Carlo analysis model, the authors evaluated the cost-effectiveness according to different scenarios (variation of ERCPs volume, Quality of Life value and post-ERCP life span). Starting from infection transmission rates lower than 1%, the use of duodenoscopes with disposable distal ends were the most cost-effective approach. Notably, the use of SUDs resulted as the second-best approach from a cost-utility standpoint, when the costs of infection transmission were considered.

### 3.3. Environmental Impact of Single-Use Duodenoscopes

Endoscopic procedures have been identified as the third largest contributor to medical waste in hospitals, after intensive care and the pediatric department. A cross-sectional study of all endoscopies performed at two USA academic medical centers, with low and high endoscopy volumes, calculated the average disposable waste (excluding waste from reprocessing) generated during one endoscopic procedure. Then, it estimated the impact of the shift from reusable to SUDs, taking into account also the reprocessing waste. Each endoscopy generated 2.1 kg of disposable waste (46 L volume), with 9% recycled. Therefore, the estimated total waste generated during all endoscopic procedures in USA is 38,000 metric tons, which would cover 117 soccer fields. The shift to the SUDs, accounting for the reprocessing, would increase the waste by 40% [34,35]. Nguyen et al. [36] performed a life-cycle assessment of SUD and reusable duodenoscopes. They compared three types of duodenoscopes: conventional reusable duodenoscopes (TJF-Q180V; Olympus, Center Valley, PA, USA), a reusable duodenoscope with disposable endcaps (TJF-Q190V; Olympus), and a SUD (Exalt Model D; Boston Scientific, Natick, MA, USA). The authors evaluated all the steps of the scope during its lifetime, from manufacturing to its use and its disposal, including reprocessing for reusable duodenoscopes. They concluded that the CO_2_ emissions related to SUDs is 24–47 times greater than that of reusable ones, with manufacturing accounting for over 90% of the GHG emissions.

### 3.4. Alternative Strategies to Single-Use Duodenoscopes to Minimize Infections

Apart from the disposable duodenoscopes, some other tools have been developing to decrease the duodenoscope-related infections. A disposable cap for the duodenoscopes is nowadays widely used and novel reprocessing techniques have been evaluating.

#### 3.4.1. Disposable-Cap Duodenoscopes

The duodenoscope-cap prevents tissue injury from the metal edges of the endoscope distal tip, but makes manual cleaning and reprocessing of the elevator mechanism and other components of the distal end challenging, allowing for biofilm formation and persistent bacterial contamination. As such, the elevator mechanism of duodenoscopes is widely considered the main contributing factor to duodenoscope-transmitted infections [29]. Five disposable-cap duodenoscopes have been approved so far. The models are listed in Table 1. Of note, ED34-i10T2, designed by Pentax Medical, has a disposable elevator mechanism incorporated.

Data on the effectiveness and safety of distal end caps are emerging. A randomized prospective study evaluated the contamination rate of 108 duodenoscopes (ED-580XT; Fujifilm) with two different reprocessing protocols: reprocessing with the distal cap detached compared with reprocessing with the removable cap still attached. A significant reduction in bacterial contamination after high level disinfection has been described in the end cap detached group (37.0% vs. 75.9%; *p* < 0.001; relative risk 0.49, 95% confidence interval (CI) 0.33–0.71) [37]. The same authors conducted a randomized non-blinded trial evaluating the difference in the proportion of bacterial contamination in 200 duodenoscopes with detachable (TJF-Q190V; Olympus) versus 200 duodenoscope with fixed distal caps (TJF-Q180V; Olympus) after manual cleaning and high level disinfection. The analysis revealed a significantly lower rate of bacterial contamination in duodenoscopes with disposable distal caps after manual cleaning (14% vs. 7%, *p* = 0.02). After high level disinfection, the rate of potential bacterial contamination in duodenoscopes with disposable caps had a trend to be lower without reaching statistical significance (4% vs. 1.5%, *p* = 0.13) [38].

In a multicenter randomized clinical trial conducted in Canada (ICECAP Trial) that included 518 patients undergoing ERCP of various procedural complexity, duodenoscopes with disposable elevator caps (ED34-i10T2; PentaxMedical, Mississauga, Canada) reduced persistent microbial contamination following high level disinfection (relative risk 0.34, 95% CI 0.16–0.75), compared with standard scopes (ED34-i10T; PentaxMedical). Importantly, they maintained a non-inferior technical performance (technical success, 94.6% vs. 90.7%, *p* = 0.13) and similar safety outcomes [39]. Furthermore, as mentioned before, the use of a duodenoscope with a disposable end-cup has been supported by cost-utility analysis, with a lesser environmental impact [33]. However, end cap disposable duodenoscopes may decrease but not completely eliminate the risk of infection transmission compared to fully disposable duodenoscopes. Indeed, an analysis of post-marketing surveillance data from the FDA Manufacturer and User Facility Device Experience (MAUDE) database revealed that the most frequently reported device-related issue among duodenoscopes with detachable caps, was bacterial contamination (53 reports) with a wide variety of organisms including CRE. The other concerns were related to use-of-device problems (31 reports), detachment/separation of device problems (25 reports), and crack/dent in material (16 reports). These device malfunctions are potential causes of tissue damage, perforation, hemorrhage, and increasing of anesthesia and procedure time. Future post-marketing surveillance studies and real-life patient analyses are needed to clarify the benefit of these devices, especially in reducing bacterial contamination [40]. Another possible disposable alternative is the ScopeSeal by GI Scientific LLC, which is a sterile, single-use endoscopic shield to cover the distal end tip of the duodenoscope. It prevents both contamination of the duodenoscope from outside bacteria and contamination of the environment from a contaminated duodenoscope. However, data on efficacy and safety are still lacking.

#### 3.4.2. Reprocessing Techniques

Novel technical applications and reprocessing techniques are under discussion to solve or at least reduce the infection related to duodenoscopes. Some of them are: the bioburden assay, which is a rapid and low-cost test that can detect the presence of organic soil by detecting biomarkers such as protein, hemoglobin or ATP [41]. The cavitation, a phenomenon in fluid dynamics where microbubbles generated in a fluid through ultrasound interact with biofilm and collapse, leading to bacterial damage and biofilm disintegration. This method does not have disinfecting properties, but it can potentially be used prior to HLD for removal of biofilm and possibly increase the effectiveness of disinfection [42]. Moreover, the methylene blue photodynamic therapy is a method that uses laser light to induce reactive oxygen species in a methylene blue solution with bactericidal effect. Lastly, the electrical current plasma-activated argon gas is directed through the duodenoscope channel to induce bactericidal reactive oxygen and nitrogen species [43].

## 4. Discussion

The duodenoscope-related infections are a major concern in medicine and GI endoscopy. They lead to high morbidity and mortality for sepsis, especially in fragile patients. Moreover, the recent COVID-19 pandemic made it clear that the risk of patient to patient viral transmission can be very high in healthcare facilities and it should be minimized. The need for a new system that can reduce the impact of infections is thus mandatory and cannot be further delayed. SUD seem to be the right tool to minimize the problem. Their safety and efficacy in performing ERCP is proven in many studies. However, the introduction of SUDs carries many issues.

**Figure 2:**
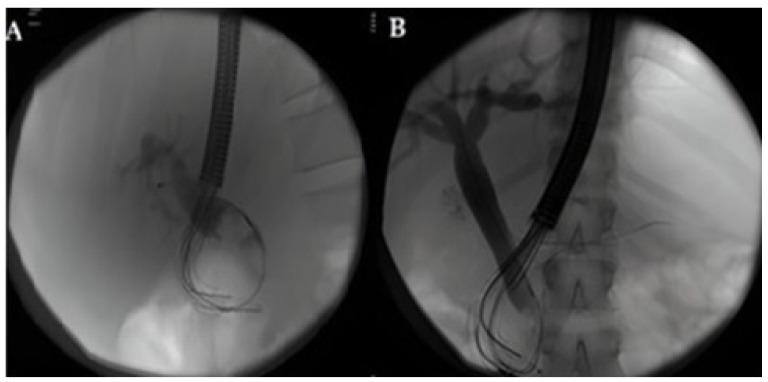
(**A**,**B**): X-ray image of an ERCP procedure with Ambu Single-Use Duodenoscope. Two different cases of elderly patients with cholangitis and many comorbidities. They had stones in the main bile duct that were successfully removed with basket.

**Figure 3:**
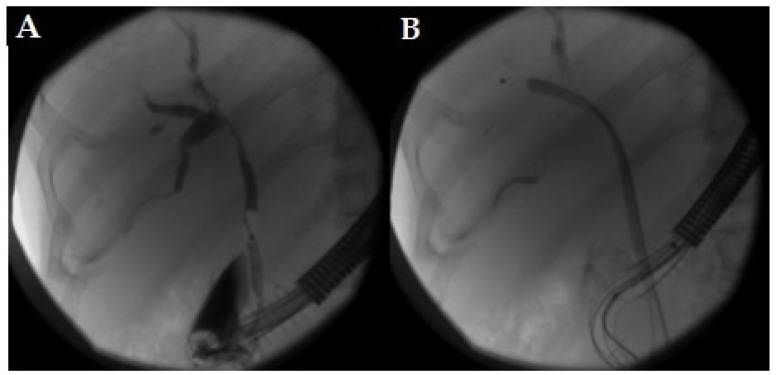
(**A**,**B**): X-ray image of an ERCP procedure with Ambu Single-Use Duodenoscope. The patient had a Klatskin tumor. The main bile duct was successfully cannulated (**A**) and a metal stent was delivered without any technical problems (**B**).

**Figure 4:**
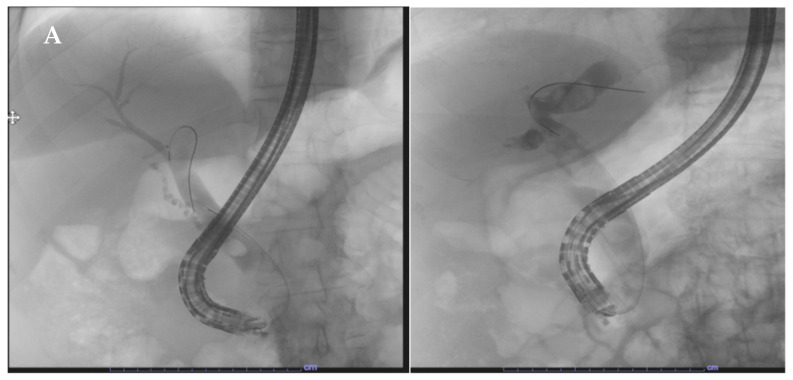

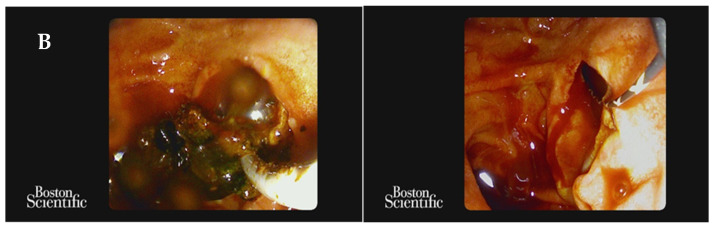
(**A**,**B**): X-ray image (**A**) and endoscopic view (**B**) of ERCP procedures and stones removal with Exalt D Single-Use Duodenoscope in a fragile patient prone to develop infection.

Firstly, the absence of randomized clinical trials and comparative large studies with reusable duodenoscopes reduces the quality of the evidences of the available studies. Moreover, the appropriateness of the use of the disposable endoscopes is a very new topic and guidelines are still lacking. Both the best clinical setting and the type of patients suitable for an appropriate choice of SUD is not well-defined by the experts. Still, there is poor evidence regarding the precise identification of the patients at risk of infection. Studies suggest that some risk factors could be independently associated with an increased risk of CRE transmission by duodenoscopes, such as biliary stenting, diagnosis of cholangiocarcinoma or recent antibiotic therapy [9]. Again, other factors seem to be associated with an increased risk of infections: more than two previous periods as inpatients in the last three months; patients admitted to Intensive Care Unit (ICU); patients with benign/undetermined biliary tract stenosis (primary sclerosing cholangitis (PSC), autoimmune cholangitis) [20,44,45]. Thus, SUDs seem to be a good choice when facing fragile patients with many comorbidities or patients with a high risk of being CRE carriers (Figure 2, Figure 3 and Figure 4). Urgent endoscopy could also be a good setting for the use of SUD, especially when reusable duodenoscopes are not available or when the risk of the infection cannot be evaluated. Moreover, the lack of a systematic culturing of scopes could also be an optimal indication for the use of SUD. Nevertheless, no definitive guidelines are available for wisely choosing the appropriate patients for ERCP with SUDs.

Regarding the economic impact of SUDs on the market, when introducing a new tool, the amplitude of the development and its impact on clinical practice depends not only on its efficacy and safety, but even from its costs and its financial impact on healthcare systems. SUD have been a breaking-trough concept for endoscopy and thus several studies have focused on the efficacy and safety of SUD for standard ERCP. More recent studies, on the other side, are evaluating the SUD financial burden, comparing its cost-effectiveness with other approaches (e.g., HLD or partially disposable duodenoscopes). They tried to highlight the specific settings that yield the best balance between efficacy and costs. Unfortunately, most studies focusing on the cost-effectiveness of SUD are conducted in USA. They are therefore based on the American medical system perspective [32], thus reducing the external validity of their conclusions to the rest of the world. The present early stage of their introduction on the market does not allow a large economic evaluation for each health system and it seems that many factors should be evaluated in the next years: the difference of health systems, the difference of the ERCP volumes of the centers, the absence of accurate comparison with other tools, such as cap-disposable duodenoscopes or new reprocessing techniques. In conclusion, a “fits-for-all” analysis of the cost-effectiveness of SUDs is very difficult due to the uncertainties of several variables involved in evaluating the overall costs of a procedure. Indeed, many variables exist: the inclusion of direct and indirect costs of a hypothetical adverse event (e.g., infection transmission) and the different reimbursement parameter of each national healthcare system. Nonetheless, most of the literature stressed that the cost utility of SUD depends mainly on the ERCP volume, the estimated rates of duodenoscope transmitted infection and the susceptibility to infections of the target patient population. Interestingly, different studies reported that the rate of duodenoscope-related infections and their economic burden may be deeply underestimated, as all the available data are elaborated only from captured outbreaks [4,46]. SUDs could be particularly cost-effective in specific settings of patients who are prone to develop infections. Even small volume centers that may not write-off the initial expenses of standard duodenoscopes may benefit from partly switching to SUDs. However, future studies are needed to better understand the economic burden of SUDs, their cost-utility and the specific settings in which a transition to SUDs could be convenient and advisable. Moreover, the risk of infection transmission of SUDs is 0% when considering the patient to patient transmission, but it is still present when considering the transmission from the patient’s GI tract to their own biliary tree.

Regarding the environmental impact of SUDs, it is still up for debate. Since SUDs are intended for single use, they generate a large amount of medical waste, including plastic, metals, and other materials, which must be appropriately disposed. This can contribute to the already significant problem of medical waste disposal, which can have serious environmental consequences. The questions arising from the latter observation are whether the disposable duodenoscopes result in a higher environmental impact compared to the reusable ones, and whether the benefits of reducing the risk of infection could outweigh the environmental harm. As it is a new field of research and development, little evidence is available on these topics. Undeniably, the healthcare sector plays a role in climate change and its devastating effects, including an increase in extreme weather events, heatwaves, wildfires, changes in vector ecology, and the spread of infectious diseases. Studies indicate that toxic pollutants produced by the health system in the United States resulted in the loss of 614,000 disability-adjusted life years in 2013. Greenhouse gas (GHG) emissions were a significant contributor to this health burden and were primarily generated by three sources: direct emissions from hospitals (such as boilers and medical gases), indirect emissions from the electricity purchased by hospitals, and supply chain emissions arising from the production of goods (such as electricity for manufacturing, truck transportation, organic chemicals, and waste management). However, electricity consumption by hospitals and supply chain activities remains the primary sources of carbon emissions within the US healthcare system, accounting for 29% of total emissions in 2018 [47]. Importantly, the expected drop in infections related to the endoscopy is also a great challenge of the single-use duodenoscopes and the balance between the harm and the gain in health is very difficult to calculate at this early stage. Surely, the impact of single-use duodenoscopes on the environment is a main point to take into consideration in the coming years, especially due to the huge climate change that the world is facing.

Last but not least, as already reported, data on the effectiveness and safety of distal end caps are emerging. Cap disposable duodenoscopes may significantly decrease the risk of bacteria and virus transmission, compared to fully reusable duodenoscopes. Their use is nowadays large and it could also be considered as a valid alternative to SUDs in patients at risk of infection. Evidence reported that among many strategies to minimize the risk of duodenoscope-related infection—single high-level disinfection, double high-level disinfection, ethylene oxide sterilization, culture and hold duodenoscopes with disposable end caps, and single-use duodenoscopes—using duodenoscopes with disposable end caps was the most preferred strategy [33]. In particular, disposable end cap duodenoscopes had superior in cost-utility compared to fully disposable duodenoscopes at typical rates of infection transmission expected in endoscopy practice. However, comparative studies between SUDs and cap disposable duodenoscopes are lacking. It would be interesting a large evaluation of the infection reduction of cap disposable scopes versus SUD and an economic and environmental evaluation of the cost–benefit of both the technologies.

## 5. Conclusions

In conclusion, SUDs seem to be a novelty destined to drop the ERCP-related infection and to offer a safer treatment to patients. The complete switch from reusable duodenoscopes to SUDs seem to be difficult to realize to date, and not immediately applicable. The main issue is the absence of large randomized clinical trials that assess the SUDs’ performance, safety and their economic and environmental impact in comparison with reusable ones. Moreover, the absence of guidelines and clear indications about the categories of patients that could benefit of SUDs creates a great heterogeneity among the hospitals and the choices of the endoscopists. To date, cap-disposable duodenoscopes seem a good alternative to SUDs to decrease the infection rate in ERCP procedures, and seem to be the best choice in terms of economical evaluation, considering the limited available data. Thus, large comparative studies between cap-disposable scopes and SUD are needed in terms of infection risk and cost–benefit balance.

## Figures and Tables

**Table 1:** FDA approved SUD and cap-disposable duodenoscopes.

Company and Type	Device
Single-Use Duodenoscope Ambu Innovation GmbH	Ambu Duodeno System
Boston Scientific Corporation	Exalt Model D Single-use duodenoscope, Exalt Controller
Cap-disposable duodenoscope Fujifilm Corporation	Fujifilm Duodenoscope Model Ed-580xt
Hoya Corporation Pentax Tokyo Office (Pentax of America, Inc.)	Pentax Medical ED34-I10T1 Pentax Medical Video Duodenoscope ED34-I10T2 2019
Karl Storz Endoskope	Albarran module

## Data Availability

No new data were created for this study.
